# Mindfulness and Leadership: Communication as a Behavioral Correlate of Leader Mindfulness and Its Effect on Follower Satisfaction

**DOI:** 10.3389/fpsyg.2019.00667

**Published:** 2019-03-29

**Authors:** Johannes F. W. Arendt, Armin Pircher Verdorfer, Katharina G. Kugler

**Affiliations:** ^1^Department of Psychology, Ludwig-Maximilians-Universität München, Munich, Germany; ^2^TUM School of Management, Technical University of Munich, Munich, Germany

**Keywords:** leadership, mindfulness, communication, mindfulness in communication, listening, leader–follower relationship

## Abstract

In recent years, the construct of mindfulness has gained growing attention in psychological research. However, little is known about the effects of mindfulness on interpersonal interactions and social relationships at work. Addressing this gap, the purpose of this study was to investigate the role of mindfulness in leader–follower relationships. Building on prior research, we hypothesize that leaders’ mindfulness is reflected in a specific communication style (“mindfulness in communication”), which is positively related to followers’ satisfaction with their leaders. We used nested survey data from 34 leaders and 98 followers from various organizations and tested mediation hypotheses using hierarchical linear modeling. Our hypotheses were confirmed by our data in that leaders’ self-reported mindfulness showed a positive relationship with several aspects of followers’ satisfaction. This relationship was fully mediated by leaders’ mindfulness in communication as perceived by their followers. Our findings emphasize the potential value of mindfulness in workplace settings. They provide empirical evidence for a positive link between leaders’ dispositional mindfulness and the wellbeing of their followers, indicating that mindfulness is not solely an individual resource but also fosters interpersonal skills. By examining leaders’ mindfulness in communication as an explanatory process, we created additional clarification about *how* leaders’ mindfulness relates to followers’ perceptions, offering a promising starting point for measuring behavioral correlates of leader mindfulness.

## Introduction

In the last decades, the construct of mindfulness, an open, non-judging awareness of the current experience (Baer, 2003), has received growing attention in psychological research (for overviews see Brown et al., 2015; Creswell, 2017; Good et al., 2016). While the bulk of research on mindfulness has been conducted in the field of health sciences, less attention has been devoted to the work context. In recent years, however, a number of researchers have started to explore whether, how, and to what degree individuals can benefit from mindfulness in the work environment (for an overview see Good et al., 2016). This research has mostly focused on positive *intrapersonal* effects (i.e., effects within individuals) of mindfulness, for instance on employee wellbeing (Roche et al., 2014; Schultz et al., 2014; Reb et al., 2015a; Malinowski and Lim, 2015), emotion regulation (Hülsheger et al., 2013), psychological detachment from work (Hülsheger et al., 2014), and job performance (Dane, 2011; Dane and Brummel, 2013; Reb et al., 2015a), while studies on *interpersonal* effects (i.e., effects between individuals) are just beginning to emerge. This is reflected in the recent call by Good et al. (2016), stating that “research in neuropsychology, cognitive psychology, medicine, and related disciplines has laid the groundwork for developing and testing theory about how mindfulness might affect relational processes, such as teamwork and leadership, but management scholars have not yet seriously undertaken that challenge” (p. 127).

The present research attempts to address this call by focusing on the relationship between leaders and followers. While some theoretical work has addressed the potential role of mindfulness in the leadership process (e.g., Glomb et al., 2011; Sauer and Kohls, 2011; Sauer et al., 2011), empirical evidence is scant. Two studies reported by Reb et al. (2014) provided first evidence for a positive effect of leaders’ mindfulness on follower wellbeing and work performance. Similarly, Reb et al. (2018) found a positive relationship between leader mindfulness and followers’ reports of leader–member (LMX) quality. These studies, however, did not investigate how leaders’ mindfulness manifests in actual behaviors that influence their interactions. Thus, the specific mechanisms and “behavioral correlates” of leaders’ mindfulness as well as its effects remain unclear and are yet to be explored. Against this backdrop, the main purpose of the present research is to enhance our understanding of the underlying behavioral mechanisms linking leaders’ mindfulness to follower outcomes. We adopt a communication-centered view of leadership (de Vries et al., 2010; Fairhurst and Connaughton, 2014; Ruben and Gigliotti, 2016) and propose that leaders’ mindfulness relates to a specific communication style of leaders that we term “mindfulness in communication.” This communication style, in turn, is assumed to predict followers’ interaction satisfaction as well as their overall satisfaction with the leader.

Overall, there are several reasons why exploring the interpersonal effects of leaders’ mindfulness in more detail is enriching and worthwhile, thus offering valuable contributions to the pertinent literature. First, we contribute to the emerging literature on mindfulness at work, especially with regard to its interpersonal qualities. By examining the relationship between leader mindfulness and actual leader behaviors, we identify interesting and compelling relations that help us better understand the mechanisms that carry the effects of leader mindfulness to employees (Good et al., 2016; Sutcliffe et al., 2016). Second, we contribute to the literature on leader communication by exploring the assumption that mindfulness may serve as a determinant of a more successful communication style. Related to this, by introducing a behavioral measure of mindfulness in communication, we add to a more thorough understanding of effective leader communication repertoires (Sager, 2008; de Vries et al., 2010).

### Mindfulness

Given the heterogeneous strands of research on mindfulness, definitions of the construct vary. However, most definitions share two key elements: *attention* and *acceptance* (Bishop et al., 2004). Specifically, with regard to these key elements, mindfulness means fully paying attention to what is happening in the present moment, both to internal (i.e., emotions and thoughts) and external stimuli with an open, non-judging attitude. Accordingly, Baer (2003) defined mindfulness as “the non-judgmental observation of the ongoing stream of internal and external stimuli as they arise” (p. 125). At this point, however, it is important to emphasize that the non-judgmental aspect of mindfulness does not imply that mindful individuals do not make any judgments at all. It rather refers to the ability to pay attention and to equanimously observe the current experience instead of getting carried away by the own immediate reactions (Dreyfus, 2011). Thus, the non-judgmental attitude should not be misunderstood as being indifferent or aloof, but it describes a form of equanimity which allows individuals to act cautiously instead of react reflexively. In connection with this, a key process of mindfulness, postulated by various scholars, is the ability to mentally “step back” from one’s own experiences which allows an individual “to observe rather than to identify with thoughts and emotions” (Hülsheger et al., 2014, p. 2). This process has been labeled as *reperceiving* (Shapiro et al., 2006) or *decentering* (Hayes et al., 2004), both referring to a shift of perspective leading to the experience of thoughts and emotions as transient mental states and not as aspects of the self.

The conceptual roots of mindfulness are usually ascribed to centuries-old eastern and Buddhist contemplative traditions (Baer, 2003; Brown and Ryan, 2003) and a large body of research is still influenced by a Buddhist understanding of mindfulness. Some scholars (e.g., Dreyfus, 2011; Grossman, 2011; Quaglia et al., 2015; Purser and Milillo, 2015) even doubt whether it is suitable, in general, to investigate mindfulness detached from mindfulness practice and its cultural roots. They object that the current approaches in Western psychology and the conceptualization of mindfulness as a bare, non-judgmental awareness of the current experience do not fully live up to the true nature and complexity of the “original” Buddhist concept of mindfulness (for a reply to Grossman, 2011, see Brown et al., 2011). However, this criticism is countered by a growing body of research that views mindfulness as “an inherent human capacity” (Kabat-Zinn, 2003, p. 146) varying between and within individuals (Brown et al., 2011), which can be investigated detached from Buddhism and mindfulness practice (Brown and Ryan, 2004; Brown et al., 2007, 2011). Related to this, it is helpful to know that mindfulness has been studied from both a state- and a trait-perspective, depending on the research focus (Hülsheger et al., 2014). Scholars have used the term *state mindfulness* for the extent to which an individual is paying attention to what is happening in the present moment with an open, non-judging attitude. At the same time, however, research has consistently recognized that the average frequency and intensity with which individuals experience states of mindfulness varies between individuals, suggesting that there is a trait-like tendency toward mindful states (Brown and Ryan, 2003; Glomb et al., 2011; Hülsheger et al., 2013; Jamieson and Tuckey, 2017; Mesmer-Magnus et al., 2017). Accordingly, it is well-established in the pertinent literature to use the terms *dispositional mindfulness* or *trait-mindfulness* to describe this tendency (e.g., Chiesa, 2013; Good et al., 2016; Mesmer-Magnus et al., 2017) and to employ self-report measures for its assessment (Bergomi et al., 2013; Sauer et al., 2013b). Longitudinal studies revealed a significant and positive association between individuals’ overall dispositional mindfulness scores and state mindfulness scores, assessed in their regular day-to-day lives (Brown and Ryan, 2003; Hülsheger et al., 2013, 2014, 2015). Also, there is solid evidence that dispositional mindfulness can be increased by mindfulness practice such as mindfulness meditation or other mindfulness-based interventions (for meta-analytic evidence see Eberth and Sedlmeier, 2012; Cavanagh et al., 2014; Quaglia et al., 2016).

Against this background, the focus of our study is on self-reported dispositional mindfulness and its effects on the outcome variables under investigation. This is in line with previous research on leader–employee relations that “reflect experiences and behaviors over extended time periods, making a state-level approach less suitable” (Reb et al., 2018, p. 2). For the sake of simplicity, we herein use the term mindfulness (or mindful leaders) to describe those higher in self-reported dispositional mindfulness.

### Mindfulness and Leadership

In organizational research, scholars have mainly focused on intrapersonal effects of mindfulness and mindfulness-based interventions (e.g., Hülsheger et al., 2014, 2015; Roche et al., 2014; Shonin et al., 2014), whereas the effects of mindfulness on interpersonal interactions and relationships have been largely neglected (Good et al., 2016). However, it is the interpersonal relationship between the leader and the followers which is at the core of leadership (Northouse, 2013) and thus, especially interesting for research in this area. Yet, only a few theoretical papers have so far addressed the role of mindfulness in leader–follower relationships (Glomb et al., 2011; Sauer and Kohls, 2011), examining the possibility that mindfulness generally helps leader better deal with various demands of leadership. Yet, as mentioned at the outset of this article, empirical evidence in this area is at a rather early stage. In two studies, Reb et al. (2014) found that followers of leaders scoring high on dispositional mindfulness reported higher levels on different aspects of wellbeing and job performance. These studies identified psychological need satisfaction as a mediator in the relationship between self-reported dispositional mindfulness of the leader and follower outcomes. In a similar vein, in a very recent study, Reb et al. (2018) found a positive relationship between leader mindfulness and follower reports of LMX quality. This effect was mediated by reduced employee stress and perceptions of increased interpersonal justice. Importantly, psychological need satisfaction and reduced stress describe internal states of followers. Also, while interpersonal justice refers to perceived fair treatment, Reb et al. (2018) conceptualized and measured it as a rather subjective assessment and therefore, the question of what *behaviors* mindful leaders actually show remain largely unanswered in their studies.

### Leadership and Communication

In the present study, we expand prior research by investigating how leader mindfulness may be reflected in visible leader behaviors, which, in turn, are expected to positively affect employee satisfaction. Specifically, we draw on a communication perspective of leadership (Fairhurst and Connaughton, 2014; Ruben and Gigliotti, 2016) and propose that the answer can be partly found in how leaders communicate, as perceived by followers. In fact, leadership is inherently about influencing others (Yukl, 2010; Northouse, 2013) and accordingly, the notion that communication is central to leadership is well established in leadership research. Organizational behavior researchers typically study leadership communication from a transmissional perspective [see Fairhurst and Connaughton (2014) for a detailed discussion of this issue], describing it in terms of “the intentional creation of messages with particular influence outcomes in mind” (Ruben and Gigliotti, 2016, p. 470). In particular, approaches of transformational and charismatic leadership have portrayed effective leaders as effective communicators, who convey an inspiring vision and high performance expectations to their followers (Antonakis, 2012). At the same time, research in this field has emphasized that the nature of leadership as an influencing process is neither leader-centric nor follower-centric but relational (Uhl-Bien et al., 2012; Fairhurst and Connaughton, 2014). That means that communication in leadership is not adequately conceptualized as a linear process, in which intentional messages simply flow in a straight and predictable line from the leader to the follower. Rather, leaders and followers continuously interact and communicate reciprocally. This is also reflected in the literature on LMX quality. Whereas high-quality relationships are characterized by cooperative communication, lower quality relationships reflect more traditional supervision with one-sided top-down communication, including higher levels of interpersonal dominance and autocratic decision-making (Sparrowe et al., 2006). That being said, and given the inherent power differential associated with most leader–follower relationships (Dulebohn et al., 2012), the way leaders shape their communication with followers is pivotal for fostering relationship quality and relevant work outcomes, such as followers’ satisfaction, commitment, and performance (Penley et al., 1991; Fix and Sias, 2006; Abu Bakar et al., 2010). Based on this, we below develop the argument that mindfulness enables leaders to engage in a more successful communication style.

### Mindfulness and Leader Communication

Following de Vries et al. (2010) a leader’s communication style represents a “distinctive set of interpersonal communicative behaviors” (p. 368). Mindfulness, with its inherent focus on being present and non-judgmental, seems particularly suitable for promoting the quality of communication. Specifically, we assume mindfulness to be related to specific communication behaviors that we term *mindfulness in communication*. Drawing on the mindfulness literature, we propose that mindfulness in communication consists of three facets: (a) being present and paying attention in conversations, (b) an open, non-judging attitude, and (c) a calm, non-impulsive manner. These features inherently reflect interpersonal attunement (Parker et al., 2015) and thereby fit well with a relational view of communication in leadership, in which influence is understood to result from interaction (Ruben and Gigliotti, 2016). In what follows, we provide a detailed rationale for our assumption that leader dispositional mindfulness is reflected in these three facets of mindfulness in communication.

First, an inherent element of mindfulness is presence, referring to “the bare awareness of the receptive spaciousness of our mind” (Siegel, 2007, p. 160). With this, the link to communication is straightforward: bare awareness, or the conscious and “direct experience of here-and-now sensory information” (Parker et al., 2015, p. 226) is expected to result in a high level of attention in interactions. Individuals who are able to focus on the immediate *now* are not distracted by thoughts and rumination concerning past or future events. This, in turn, is an important prerequisite for effective listening (Brownell, 1985). The importance of listening for effective leader–follower communication has been stressed by several scholars (Bechler and Johnson, 1995; Johnson and Bechler, 1998; Alvesson and Sveningsson, 2003; van Vuuren et al., 2007). In a survey of van Vuuren et al. (2007), for example, listening was shown to be the second most important factor of leader communication style for follower commitment. Furthermore, there is empirical evidence that careful listening is associated with transformational leadership (Berson and Avolio, 2004) and effective interpersonal influence (Ames et al., 2012). Also, a qualitative study conducted by Alvesson and Sveningsson (2003) revealed that leaders themselves consider listening a central feature of their role. Empirical support for the notion that leaders’ dispositional mindfulness may translate into improved listening skills comes from several studies linking dispositional mindfulness and mindfulness trainings to reduced rumination and improved attentional performance (e.g., Chambers et al., 2008; Jensen et al., 2012; Flook et al., 2013; Roeser et al., 2013). Moreover, an intervention study conducted by Beckman et al. (2012) showed that physicians who participated in a communication training, which included mindfulness meditation, reported that mindfulness improved their abilities to be attentive and to better listen to their patients.

The second rationale for linking leader mindfulness to leader communication style is based on the second essential feature of mindfulness, namely acceptance. Acceptance refers to “being experientially open to the reality of the present moment” (Bishop et al., 2004, p. 233), “without being swept up by judgments” (Parker et al., 2015, p. 226). This non-judgmental, present-centered awareness may help leaders to keep an open mind in interactions with their followers and to be open to other perspectives and opinions without rashly evaluating and categorizing incoming information. By paying attention in a non-judgmental manner, mindful individuals (i.e., leaders) are better able “to retain information and thus see their true significance rather than being carried away by their reactions” (Dreyfus, 2011, p. 47). In this understanding, mindful leaders are not free of making judgments and evaluations. However, before doing so, they give their followers the opportunity to fully communicate their message and let their attention not be influenced by automatic reactions and rash interpretations.

The third rationale refers to research linking mindfulness to effective emotion regulation (Chambers et al., 2009; Heppner et al., 2015). Accounting for this effect, scholars have consistently referred to the process of *reperceiving* (Shapiro et al., 2006) or *decentering* (Hayes et al., 2004) and argued that mindfulness permits individuals to disidentify from their emotions and experience them as transient cognitive events rather than aspects of their self and thus as less threatening. There is robust empirical evidence that mindfulness is associated with lower levels of negative affect and higher levels of positive affect (Baer et al., 2006; Luberto et al., 2014; Pepping et al., 2014; Prakash et al., 2015). Accordingly, mindfulness enables leaders to better deal with negative affective states and stressful events. In terms of communication, better emotion regulation should be reflected in an increased ability to maintain composure in tense situations instead of being overwhelmed by emotions.

Empirical support for the assumed relation of mindfulness and communication behavior comes from marital and family research (O’Kelly and Collard, 2012). Several studies in this area found a positive relationship between mindfulness and outcomes pertaining to communication quality among couples, such as perspective taking and empathic concern (Block-Lerner et al., 2007), constructive conflict (Barnes et al., 2007), and mutual acceptance (Carson et al., 2004). Moreover, Krasner et al. (2009) designed and evaluated a communication training for primary care physicians that included mindfulness meditation (see also Beckman et al., 2012). After the training, participants demonstrated improvements in dispositional mindfulness and, importantly, perspective taking when relating to patients.

Taken together, we propose that leaders’ dispositional mindfulness is positively related to specific communication behaviors (*mindfulness in communication)*, as perceived by their followers.

*Hypothesis 1*: Leaders’ dispositional mindfulness is positively related to specific communication behaviors – i.e., “mindfulness in communication.”

### Leader Mindfulness, Mindfulness in Communication, and Follower Satisfaction

In this section, we develop the argument that leaders’ dispositional mindfulness has positive effects on followers’ outcomes mediated by mindfulness in communication. Most notably, as a very proximal outcome, we explore the degree to which followers are satisfied with the communication with their leader. Thereby, we assume all three components of mindfulness in communication (i.e., paying attention, being open and non-judgmental, and a calm, non-impulsive manner) to be important for how followers perceive the communication with their leaders.

According to Thayer (1968), individuals experience satisfaction with the communication when communication is perceived as successful. Following Ruben and Gigliotti (2016), who state that, “leadership communication always has both content and relational consequences” (p. 476), successful communication refers to the quality and accuracy of information transmission (i.e., content consequences) as well as to the fulfillment of personal needs, aspirations, and expectations of the involved agents (i.e., relational consequences). Leaders’ mindfulness in communication is likely to foster followers’ satisfaction on the content level because less information gets lost between “sender” and “receiver” and the information is processed in a less biased manner. This assumption is supported by various empirical findings, linking mindfulness to increased attention focus and less attentional biases (e.g., Chambers et al., 2008; Flook et al., 2013; Roeser et al., 2013). With regard to the relational level, we follow Reb et al. (2014) and draw on self-determination theory (SDT; Deci and Ryan, 2000; Ryan and Deci, 2000), implying that leaders who communicate mindfully can help satisfy the basic needs of followers, which results in increased satisfaction (Deci et al., 2017).

The need for autonomy describes the desire to be in control of one’s environment. One way for leaders to help ensure that followers experience some level of control is to provide voice, listen attentively, and treat requests seriously (Folger and Cropanzano, 1998). By paying full attention and listening to their followers, leaders signalize that they are open to the input of their followers and are serious about what they have to say (Ashford et al., 2009). Furthermore, by showing an open and non-judgmental attitude, leaders signal that they are willing to see things from their followers’ perspective and offer them voice-opportunities (Ashford et al., 2009; Lloyd et al., 2015), which enables followers to address and openly speak about organizational problems.

In a similar vein, mindfulness in communication is likely to satisfy followers’ need of competence, which refers to feelings of growth, ability, and achievement. Specifically, through paying full attention and a high degree of acceptance and calmness, leaders show their followers that their opinion and viewpoints are regarded as important and worthwhile to consider, reflecting genuine appreciation of their strengths and unique abilities (Van Quaquebeke and Felps, 2016; Deci et al., 2017).

Finally, individuals, who have their relatedness need met, feel secure and safe in their environment and in their relationships with others. When leaders are fully paying attention with an accepting, non-judging attitude, they are likely to generate a feeling of being valued and respected in of their followers (Reb et al., 2014). Furthermore, this kind of leader communication behavior may foster a feeling of psychological safety and intimacy in their followers (Ashford et al., 2009; Lloyd et al., 2015) as well as a feeling of being cared about (Van Quaquebeke and Felps, 2016) which has empirically been linked with relatedness (Reis et al., 2000). Thus, leaders’ mindfulness in communication is likely to result in an enhancement of followers’ experience of relatedness.

Given that communication is central to leadership (Alvesson and Sveningsson, 2003; Yukl, 2010; Ruben and Gigliotti, 2017) this satisfaction is likely to correspond to an increase in overall satisfaction with the leader (Miles et al., 1996). This claim can also be deduced from theory on human affective experiences. Fully present, non-judging leaders who keep calm even in intense situations are likely to elicit positive affective reactions in their followers due to an immediate satisfaction of basic psychological needs. Reversely, non-listening, rashly judging leaders, who easily get worked up are likely to elicit negative affective reactions from their followers. According to affective events theory (Weiss and Cropanzano, 1996), such affective reactions, especially if experienced repeatedly, likely result in generalized satisfaction judgments about the leader. Notably, this notion is reflected in prior research, positioning the way leaders listen and pay attention to what employees have to say as an important facet of employees’ satisfaction with their leader (Scarpello and Vandenberg, 1987). In a similar vein, two studies by Bechler and Johnson (1995) and Johnson and Bechler (1998) showed that the evaluation of leadership skills is positively related to perceived listening skills. Taken together, we predict:

*Hypothesis 2*: Leaders’ mindfulness in communication mediates the positive relationship between leaders’ dispositional mindfulness and (a) their followers’ satisfaction with the communication with the leader and (b) the satisfaction with the leader in general.

**FIGURE 1:**
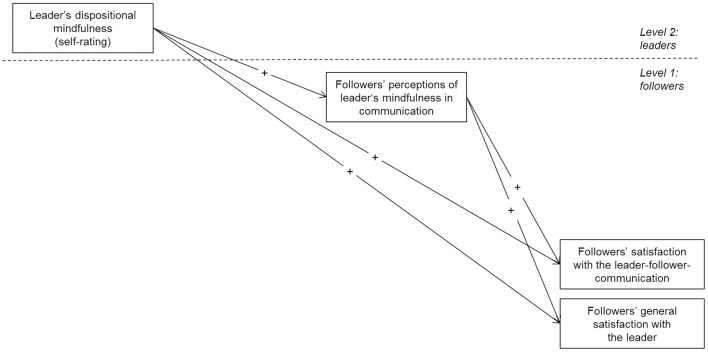
Hypothesized theoretical model.

Figure 1 shows the hypothesized theoretical model.

## Materials and Methods

We certify that the research presented in this manuscript has been conducted within the DGPs (German Psychological Society) ethical standards regarding research with human participants and scientific integrity. We adhere to the ethical standards of the DGPs, since in Germany there is no legal regulation for approval of research through a research ethics committee for the social sciences, but ethics questions are addressed within a framework by professional associations. Participants were free to not participate and to terminate participation at any time without any consequences or any loss of benefits that the subject was otherwise entitled to receive. All subjects have given written informed consent in accordance with the Declaration of Helsinki.

### Sample and Procedures

In order to test our hypotheses, we conducted a multilevel field study. Online surveys were sent to leaders as well as their followers. We assessed leaders’ self-rated dispositional mindfulness on the one hand and followers’ perceptions of leaders’ mindfulness in communication as well as followers’ self-reported satisfaction with the leader (i.e., satisfaction with the leader–follower communication and general satisfaction) on the other hand. The study had a cross-sectional design. Given this design, no causal conclusions can be drawn from our study, which has to be taken into account when interpreting the results.

Followers and their leaders were recruited from various organizations of different industries in Germany, Austria, and the German speaking part of Switzerland by using three different strategies. First, individuals from our personal and professional networks were contacted. Second, we contacted HR departments of various organizations and third, the study was advertised in social networks (mainly XING). Individuals who were interested in participating received a link to the online questionnaire in addition to instructions on how to forward a separate link to their supervisor or followers, respectively. Anonymous identification codes generated by the participants were used to match the data of leaders and followers.

A total of 351 participants (147 leaders and 204 followers) completed the questionnaires. Out of the 204 followers, 141 could be matched to 77 leaders. For 43 leaders, we received responses from one follower only; for 34 leaders, we received responses from more than one follower (ranging from two to six followers, *M* = 2.88, *SD* = 1.17). Since we were interested in a general assessment of leaders by their followers, independent of specific biases of single followers, we used only those leaders for our final analysis, for which we had responses of multiple followers (i.e., at least two followers). Thus, the final sample consisted of 98 followers nested in 34 leaders.

In our final sample, 50% of the participants were female, the average age was 37.21 years (*SD* = 9.86), and 64% of the participants had a university degree. The sample consisted of individuals from Germany (64%), Austria (28%), Switzerland (5%), and other nationalities (3%). The participants’ average tenure in the organizations was 7.93 years (*SD* = 7.52), their average weekly working hours were 42.91 h (*SD* = 10.33). Followers’ average tenure with their leaders was 3.18 years (*SD* = 3.18), the average interaction frequency between leaders and followers was 11.05 h (*SD* = 11.78) per week. Because we were interested in leaders’ mindfulness, we also assessed if they practiced mindfulness meditation in their daily lives: 10 of the 34 leaders of our final sample reported practicing some form of mindfulness meditation. However, we did not assess the amount or the specific nature of the mindfulness practice since this was beyond the scope of the present study.

### Measures

#### Dispositional Mindfulness

Leaders’ *dispositional mindfulness* was measured with the short-version of the Freiburg Mindfulness Inventory (Walach et al., 2004, 2006). The scale consists of 14 items assessing the frequency of mindful states. A sample item is “I am open to the experience of the present moment”. The items were answered on a 6-point frequency scale (ranging from 1 = *never* to 6 = *almost always*). Cronbach’s alpha was 0.90.

#### Followers’ Satisfaction With Leader–Follower Communication

In order to measure *followers’ satisfaction with leader–follower communication*, we used two items from the questionnaire for communication in organizations developed by Sperka (1997). The two items were “I am content about how the communication with my leader takes place” and “I would like to have a better communication with my leader” (reverse coded). Each follower was asked to rate their own level of satisfaction. Again, a 6-point response scale (ranging from 1 = *strongly disagree* to 6 = *strongly agree*) was employed. The reliability of this measure was estimated by using the Spearman–Brown formula (see the recommendations for the use of two-item scales by Eisinga et al., 2013), and was 0.66.

#### Followers’ General Satisfaction With Their Leaders

*Followers’ general satisfaction with their leaders* was measured with two items taken from Felfe (2006). The two items were: “My leader uses methods of leadership that are satisfying” and “My leaders works with me in a satisfactory way.” Responses were given on a 6-point scale, ranging from 1 = *strongly disagree* to 6 = *strongly agree*. The reliability of this measure was again estimated by using the Spearman–Brown formula (see the recommendations for the use of two-item scales by Eisinga et al., 2013), and was *r* = 0.84.

#### Mindfulness in Communication

Since there was no existing scale for what we call *mindfulness in communication*, we developed a new scale for this study. The purpose of the scale was to assess “behavioral correlates” of leaders’ mindfulness when communicating with followers. Followers were explicitly asked to rate their leaders’ behavior in communication situations. Below, we describe in more detail how the measure was constructed.

##### Item development and exploratory factor analysis

We first generated 14 items based on a review of the literature addressing mindfulness in leadership (Glomb et al., 2011; Sauer and Kohls, 2011; Sauer et al., 2011; Reb et al., 2014). The items addressed the following three facets of mindfulness in communication: (1) being present and paying attention in conversations, (2) showing an open, non-judging attitude during a conversation, and (3) being calm and non-impulsive during conversations, not becoming overwhelmed by emotional reactions. Second, the content validity was assessed by asking four experts (i.e., experts on mindfulness practice) to rate the items in terms of their conceptual fit. As a result of the expert rating, four items were omitted. The remaining 10 items were included in the questionnaire described above and were answered in total by 204 followers (including followers that could be matched with a leader and followers that could not be matched with a leader and were therefore not considered in the main analyses). For the analyses of the scale and the items, all followers (*N* = 204) were included. One item showed a low level of communality (communality = 0.17) and was therefore excluded. With the remaining nine items, an Exploratory Factor Analysis using a principal–axis analysis with Promax rotation was performed. The results suggested one factor with an Eigenvalue > 1 explaining 59% of the variance. The factor loadings, communalities, and standardized item-scale-correlations were satisfactory (Table 1). Therefore, the final scale consists of nine items and shows adequate reliability (α = 0.91) which could not be improved by deleting further items.

**Table 1:** Items of the scale “mindfulness in communication” including their factor loadings, communalities, and corrected item-scale-correlations.

Facets and items		Factor loadings	Commu-nalities	Corrected item-scale correlation
Being present by paying attention to the other				
	I have my supervisor’s full attention when I am speaking	0.70	0.49	0.66
	In conversations, my supervisor is impatient (R)	0.68	0.46	0.65
	My supervisor is only half-listening when I am talking (R)	0.72	0.52	0.69
Showing an open, non-judging attitude				
	In conversations my supervisor first listens to what I have to say, before forming his/her own opinion	0.81	0.66	0.76
	Before I have finished talking, my supervisor has already formed his/her own opinion (R)	0.77	0.59	0.73
	My supervisor has a preconceived opinion about many topics and holds on to this opinion (R)	0.71	0.50	0.67
Being calm and non-impulsive during conversations				
	My supervisor stays calm even in tense situations	0.72	0.52	0.68
	My supervisor gets easily worked up (R)	0.75	0.57	0.72
	When my supervisor does not like something, emotions can easily boil over (R)	0.74	0.54	0.71


*Items were assessed on 6-point Likert scale ranging from 1 = strongly disagree to 6 = strongly agree. An Exploratory Factor Analysis was calculated (principle-axis-analysis with a promax rotation) with *N* = 204 employees using SPSS. The original version of the scale, which we used in our study, was in German. For this publication, the scale was translated by two individuals, who then agreed on a final version: a native German speaker, who is fluent in English and a native English speaker, who is fluent in German.*

##### Confirmatory factor analysis

Because a new measure of mindfulness in communication was created for this study, we collected data from a separate sample to confirm the construct validity using confirmatory factor analysis (CFA). Specifically, 214 employees from various organizations in Germany completed the newly developed measure. The mean age of the participants was 33.36 (*SD* = 8.89); 47% were male and 65% had a university degree. The majority of the participants worked in the for-profit sector (69%) and the average tenure in the current position was 3.92 years (*SD* = 3.25).

We conducted a CFA using the Lavaan package in R (Rosseel, 2012) and compared the fit of two nested models. The first one was a single-factor model with all nine items loading on the same factor. The second one was a second-order factor model in which items loaded on their respective factors (i.e., presence, openness, and calmness) and the three factors loaded on a second-order latent mindfulness in communication factor. This second-order factor model showed a reasonable fit with χ^2^ = 58.65, *df* = 24, *p* < 0.001, comparative fit index (CFI) = 0.96, root mean square error of approximation (RMSEA) = 0.08, SRMR = 0.04 and was clearly preferable over the single-factor model (χ^2^ = 219.82, *df* = 27, *p* < 0.001, CFI = 0.80, RMSEA = 0.18, SRMR = 0.85; Δχ^2^ = 161.17, *df* = 3, *p* < 0.001, ΔCFI = 0.16). It should be noted that with three latent factors, the second-order model is mathematically equivalent to a first-order model (i.e., a model in which items load on their respective factors and the factors are allowed to correlate) and thus, both solutions produce identical fit statistics (Rindskopf and Rose, 1988; Hoyle, 2011). Yet, since we assumed that a common latent mindfulness in communication factor accounts for the relation between the three subscales (i.e., presence, openness, and calmness), the second-order model represents a more parsimonious and meaningful approach (Rindskopf and Rose, 1988; Chen et al., 2005). This supports the use of the combined mindfulness in communication scale (comprising the three sub-facets) in the analysis presented below (McGartland Rubio et al., 2001).

##### Discriminant validity

To examine the discriminant validity among the follower-related outcome measures that we used in our main study (i.e., perceived mindfulness in communication, satisfaction with the communication with the leader, and general satisfaction with the leader), we again used CFA. For mindfulness in communication, we used parcels as indicators (i.e., the three sub-dimensions, which is in line with the content-based algorithm of parcel building, see Matsunaga, 2008). For the two satisfaction constructs, items were used as indicators. Table 2 reports the models we tested. To compare the fit for different models, we used the chi-square difference test. However, given that chi-square tests are very sensitive to sample size and non-normality, even small differences may become statistically significant (Brannick, 1995; Kline, 2011). Thus, in line with the recommendations of Chen (2007), we also relied on the change in CFI and RMSEA. Specifically, for small samples (i.e., *N* < 300) a change of 0.005 in CFI, supplemented by a change of 0.010 in RMSEA indicates that the models are significantly different. As shown in Table 2, the proposed three-factor model (Model 1) fitted the data reasonably well and was preferable over all alternative models (Models 2, 3, and 4). Taken together, these results provide evidence that our follower reported measures captured distinct constructs.

**Table 2:** Test of measurement models.

Model	χ^2^ (*df*)	CFI	RMSEA	Δχ^2^ (*df*)	ΔCFI	ΔRMSEA
Model 1: three factors (mindcom, leadsat, and comsat)	17.96 (11)	0.99	0.07			
Model 2: two factors (leadsat and comsat treated as 1 factor)	27.63 (13)	0.97	0.09	9.66_(2)_^∗∗^	0.01	0.02
Model 3: two factors (mindcom and leadsat treated as 1 factor)	86.76 (13)	0.86	0.20	68.79_(2)_^∗∗∗^	0.12	0.13
Model 4: two factors (mindcom and comsat treated as 1 factor)	56.94 (13)	0.92	0.15	38.98_(2)_^∗∗∗^	0.07	0.09


*^∗^*p* < 0.05, ^∗∗^*p* < 0.01, ^∗∗∗^*p* < 0.001. Mindcom = mindfulness in communication, Leadsat = satisfaction with leader in general, Comsat = satisfaction with communication, Δχ^*2*^, ΔCFI, and ΔRMSEA are in comparison to the three factor model. Regarding the mindcom measure, the same pattern of differences was revealed, when items were used as indicators instead of parcels.*

## Results

### Descriptive Statistics

Correlations, means, and standard deviations of all variables are shown in Table 3 [calculated in R using *psych* (Revelle, 2016); *apaTables* (Stanley, 2015)]. Neither tenure with the leader nor perceived interaction frequency was related to our main variables^1^. In turn, leaders’ dispositional mindfulness was positively correlated with followers’ perceptions of leaders’ mindfulness in communication as well as with the two satisfaction ratings. Also, the correlations between followers’ perceptions of leaders’ mindfulness in communication and the two satisfaction measures were in the expected direction.

**Table 3:** Means, standard deviations, ICC(1), ICC(2), and correlations.

Variable	*M*	*SD*	ICC(1)	ICC(2)	1	2	3	4	5	6
Level 1 variables										
1. Tenure with leader in years	3.18	3.19								
2. Interaction frequency between leader and follower in hours per week	11.05	11.79			0.02					
3. Followers’ perception of the leaders’ mindfulness in communication	4.87	0.94	0.25	0.50	-0.02	-0.08	(0.91)			
4. Followers’ satisfaction with leader–follower communication	4.54	1.14	0.24	0.48	-0.03	0.08	0.60**	(0.66)		
5. Followers’ general satisfaction with the leader	4.00	0.89	0.29	0.54	-0.01	0.06	0.60**	0.64**	(0.84)	
Level 2 variables										
6. Leaders’ dispositional mindfulness	4.05	0.79			-0.09	-0.03	0.31**	0.32**	0.28**	(0.90)


*^∗^*p* < 0.05. ^∗∗^*p* < 0.01. Reliabilities (Cronbach’s alpha or, in case of two-item-scales, Spearman–Brown coefficient) are indicated on the diagonal in parenthesis. *N* = 98. For cross level correlations, the level 2 variable was disaggregated by assigning each member of a group the same value.*

### Analytic Strategy

Our dataset had a multilevel structure, given that we asked leaders about their level of mindfulness (i.e., independent variable on Level-2), and we asked at least two followers of those leaders about their leaders’ mindfulness in communication and their own satisfaction (i.e., mediator and dependent variable on Level-1). Thus, we first examined the nested structure of our data (Bliese, 2000; LeBreton and Senter, 2008) using R (R Core Team, 2017); *multilevel* (Bliese, 2016). First we examined the variance between the groups of followers reporting to one leader. An ANOVA showed significant differences between the groups of followers. The ICC(1), which is reported in Table 3, indicated that 24–29% of the variance resided between groups. Second, we examined the agreement within groups of followers reporting to one leader. The ICC(2), which is also shown in Table 3, indicated an agreement between 0.48 and 0.54. It is helpful to note that ICC(2) is dependent on the group size (Bliese, 1998). In our study, the average group size was 2.8 and ICC(2) values ranging from 0.48 to 0.54 correspond with Bliese’s (1998) estimates about what can be statistically expected.

To test our hypotheses within the multilevel framework, we followed the suggestions for multilevel mediations suggested by (Zhang et al., 2009). Our mediation model reflects a 2–1–1 design (Zhang et al., 2009) with leaders’ dispositional mindfulness representing the Level-2-predictor, perceived leaders’ mindfulness in communication representing the Level-1 mediator, and followers’ satisfaction ratings representing Level-1 outcomes (Figure 1). In 2–1–1 models the within-group effects and between-group effects are confounded (Zhang et al., 2009) – in our case the effect between followers of different leaders on the one hand and within followers of a particular leader on the other hand. To address this problem, Zhang et al. (2009) suggested to differentiate the between and the within group effects by inserting the mediator at both levels in the following way: At Level 1, the mediator is centered around the group mean, specifying the within-group effect. At Level 2, the mediator is aggregated for each group using the group mean in order to specify the between-group effect. In line with these recommendations, we included our mediator variable (i.e., perceived leaders’ mindfulness in communication) at both levels (i.e., group mean centered at Level 1 and aggregated for the followers of a particular leader at Level 2). Because mindfulness in communication was thought to be a characteristic of each leader, we were especially interested in the effect between followers of different leaders and thus, the effect at Level 2. Consequently, the effects within followers (i.e., Level 1) were treated as control variable “only.” Nevertheless, we report the coefficient for both effects (within-groups effects and between-groups effects). Given that we used the aggregated values of mindfulness in communication as our mediator, we calculated the *r*_wg_ statistic (James et al., 1984) to assess the appropriateness of aggregating [in addition to relying on the ICC(2) value, which was reported above]. The mean *r*_wg_ for perceived mindfulness in communication was 0.74, indicating strong interrater agreement (LeBreton and Senter, 2008).

The results of the multilevel analysis are reported in Table 4. All calculations were conducted in R using the packages: *multilevel* (Bliese, 2016), *nlme* (Pinheiro et al., 2017), *sjmisc*/*sjstats* (Lüdecke, 2017), and *reghelper* (Hudghes, 2008).

**Table 4:** Results of multilevel mediation analyses.

	Mediator: Followers’ perceptions of leader’s mindfulness in communication (aggregated; level 2)	Dependent variable 1: Followers’ satisfaction with the leader–follower communication (level 1)			Dependent variable 2: Followers’ general satisfaction with the leader (level 1)		
			
	Model 1	Model 2	Model 3	Model 4	Model 5	Model 6	Model 7
IV: Leaders’ dispositional mindfulness (level 2)	0.44 (0.16)**	0.32 (0.11)**		0.15 (0.10)	0.27 (0.11)*		0.07 (0.11)
Mediator: Followers’ perceptions of leader’s mindfulness in communication (aggregated; level 2)			0.46 (0.09)**	0.39 (0.10)**		0.48 (0.10)**	0.45 (0.11)**
Control variable: Followers’ perceptions of leader’s mindfulness in communication (group mean centered; level 1)			0.41 (0.07)**	0.41 (0.07)**		0.40 (0.07)**	0.40 (0.07)**
Explained variance *R*^2^	0.20	0.33	0.57	0.56	0.42	0.61	0.61


*^∗^*p* < 0.05. ^∗∗^*p* < 0.01. Standardized coefficients are shown; standard errors are included in parenthesis. Model 1 (i.e., single level relationship on level 2) was calculated as linear regression (*N* = 34 on level 2). Models 2–7 (i.e., multilevel relationships) were calculated as multilevel linear models with random intercepts (*N* = 34 on level 2 and *N* = 98 on level 1). For multilevel linear models an *R*^*2*^ approximation was computed based on the correlation between the fitted and observed values as suggested by Byrnes (2008).*

### Hypothesis Tests

Supporting Hypothesis 1, leaders’ dispositional mindfulness was positively related to followers’ perceptions of leaders’ mindfulness in communication (see Table 4, Model 1). In line with Hypotheses 2 and 3, we found that leaders’ dispositional mindfulness was also positively related to both followers’ satisfaction with the leader–follower communication and followers’ general satisfaction with their leaders (see Table 4, Model 2 and Model 5). In addition, we predicted that these two positive relationships were mediated by leaders’ mindfulness in communication as perceived by the followers. When followers’ satisfaction with the leader–follower communication was regressed on both mindfulness in communication and leaders’ dispositional mindfulness, the relationship with mindfulness in communication was significant whereas the relationship with leaders’ dispositional mindfulness was no longer significant (see Table 4, Model 4). Using the Monte-Carlo method for assessing indirect effects with 20,000 replications [cf., Selig and Preacher, 2008), we found that the mediation was significant (95% bias-corrected bootstrap CI (0.07, 0.52)]. Similarly, when followers’ general satisfaction with their leaders was regressed on both mindfulness in communication and leaders’ dispositional mindfulness, the relationship with mindfulness in communication was significant, whereas the relationship with leaders’ dispositional mindfulness was no longer significant (see Table 4, Model 7). Using again the Monte-Carlo method for assessing indirect effects with 20,000 replications, we found that the mediation again was significant [95% bias-corrected bootstrap CI (0.06, 0.45)]. In sum, when leaders scored high on dispositional mindfulness their followers perceived them as showing mindfulness in communication, which in turn was positively related to both followers’ satisfaction regarding their communication with the leader and satisfaction with the leader in general.

## Discussion

The aim of the present study was to enhance the understanding of whether and how leaders’ dispositional mindfulness may translate into leader behaviors that relate to follower’s perceptions and satisfaction with their leaders. We hypothesized that leaders’ mindfulness would be positively linked to specific communication behaviors, which we labeled “mindfulness in communication.” In turn, perceived mindfulness in communication was hypothesized to mediate the relationship between leaders’ dispositional mindfulness and followers’ satisfaction regarding the communication with the leader and the leader in general. The results of our empirical study supported our hypotheses.

### Contributions and Theoretical Implications

Research on mindfulness in the workplace in general and mindfulness of leaders in particular is still at an early stage and, so far, mainly consists of theoretical considerations (e.g., Glomb et al., 2011). By empirically confirming interpersonal effects of mindfulness, the results of the present research have several theoretical implications.

First, our findings provide additional evidence for a positive link between an individual’s (the leader’s) dispositional mindfulness and the wellbeing of other people (their followers), suggesting that mindfulness is not only an internal capital but also aids individuals in interpersonal relations. These results are in line with the findings of Reb et al. (2014) who first provided scientific evidence for such interpersonal effects of mindfulness in leader–follower relationships. Also, our results expand evidence that has been provided in a very recent study by Reb et al. (2018), in which leader mindfulness predicted follower reports of enhanced LMX quality. With this, our study also contributes more generally to the perennial interest in leadership research regarding the effects of leaders’ affect and emotions on their followers (for reviews see Gooty et al., 2010; Rajah et al., 2011; Walter et al., 2011). Mindfulness, which is assumed to play an important role in emotion regulation, affect, stress, and well-being (cf.,Good et al., 2016; Lomas et al., 2017; Mesmer-Magnus et al., 2017), constitutes a concept that is likely to offer new and fruitful insights for research in this area, where the emotional states of individuals have wide-ranging consequences on others.

Second, by examining leaders’ communication style as an underlying mechanism, we take a step forward in clarifying *how* leaders’ mindfulness may affect their followers. More specifically, we identify a behavioral mechanism – mindfulness in communication – which explains the interpersonal effect of leaders’ mindfulness. The high agreement of multiple followers in their ratings of the leaders’ mindfulness in communication that we found in our data (as indicated by the mean *r*_wg_) suggests that mindfulness fosters a specific communication style, which is relatively stable across situations and followers. This is in line with emerging evidence that leader mindfulness is reflected in specific leadership styles, as perceived by others. Specifically, Pircher Verdorfer (2016) conducted a study which found a positive relationship between leaders’ mindfulness and followers’ perceptions of specific servant leader behaviors, that is, humility, standing back, and authenticity. Interestingly, our notion of mindfulness in communication fits well with these features. In fact, it is plausible that leaders who are mindfully present, accepting, and calm when communication with others signal humility (e.g., being open to different views and opinions of others), the ability to stand back (e.g., not chasing recognition or rewards), and authenticity (e.g., being open about own limitations and weaknesses).

Third, our results indicate that mindfulness in communication is a useful approach that meaningfully adds to previous perspectives in the field of leader communication style. Established instruments, such as the Communication Styles Inventory (CSI) by de Vries et al. (2011) have a strong focus on how information is conveyed (e.g., in terms of preciseness or expressiveness) and whether one is generally supportive versus aggressive or tense when communication with others. With a behavioral measure of mindfulness in communication, we gain a better understanding of genuine interpersonal attunement of leaders that goes beyond the transmission of leadership messages (Parker et al., 2015). Related to this, an interesting implication of our results refers to the relationship between individual dispositions or personality traits and communication styles. de Vries et al. (2011) found support for the notion that a person’s communication style is, partially, a function of his/her personality traits. They found, for instance, expressiveness in communication to be strongly related to extraversion, while verbal aggressiveness in communication was, not surprisingly, negatively related to agreeableness. Our results add to this picture by showing that mindfulness, as a distinct disposition (Rau and Williams, 2016), likely translates into a distinct communication pattern.

### Limitations and Future Research

Despite its contributions, our work is not without limitations, offering interesting directions for future research. Most notably, due to the cross-sectional design, causal conclusions cannot be drawn from our data and the direction of the revealed effects are based on theoretical deliberations. Accordingly, alternative explanations and common underlying antecedents of all examined variables cannot be entirely excluded. For instance, research has started to explore socio-contextual factors at work, such as managerial need support, as antecedents of mindful states (Olafsen, 2016). While contextual factors may facilitate or inhibit the experience of mindful states, they may also affect the well-being and communication behavior of leaders and followers. Hence, future research would benefit from using longitudinal data and controlling for more context variables. This would also permit to shed further light on potential moderating effects. For instance, it is plausible that the beneficial effects of mindfulness in communication may best unfold in fast-paced and volatile high performance contexts, where the quality of leadership communication is particularly important for organizational adaption and functioning (Uhl-Bien et al., 2007). In contrast, in highly bureaucratic organizations with strict regulations and protocols for decision-making, communication is usually organized and formal and thus, mindfulness may be less relevant.

A second limitation refers our sample size, in that we were able to recruit, on average, only a few followers per leader. While our sample size is in line with similar studies in this field, we nonetheless hope that future studies will address this limitation and gather more data from multiple raters assessing mindfulness in communication. Importantly, high agreement among multiple raters will further corroborate our notion of mindfulness in communication as a stable communication pattern.

A third issue, one that is both a limitation and, we believe, a strength, refers to our mindfulness in communication measure, which we developed for this study. It is a strength because it allowed us to capture very proximal behavioral correlates of core aspects of mindfulness in a person’s communication behavior, while similar measures in this field tend to be much wider. For instance, measures of active listening or general communication style typically focus on being generally sensitive to the feelings and concerns of others (Drollinger et al., 2006; Bodhi, 2011; de Vries et al., 2011). Also, such measures usually include skills pertaining to information processing (i.e., remembering, summarizing, and clarifying points) and responding (i.e., asking for feedback, nonverbal signals). At the same time, however, given the constitutive nature of our work, the construct of mindfulness in communication requires further exploration and validation. Although we substantiated the psychometric properties of our newly developed measure in a separate sample, there remains room for further scrutiny with regard to its nomological network as well as its discriminant, convergent, and predictive validity. Concretely, it would be useful in future research to test our measure against the above mentioned measures of active listening (Drollinger et al., 2006) and interpersonal communication style (de Vries et al., 2010). Such studies would benefit from considering additional, more diverse outcomes, at both the individual and the interpersonal level. In terms of individual outcomes, it would be particularly fruitful to capture followers’ basic need satisfaction, as we used this in our theoretical framework but did not include it in our measurement strategy. At the interpersonal level, it would be interesting to see whether mindfulness in communication has a unique effect on the relationship quality between leaders and followers, reflected in LMX and trust (Dulebohn et al., 2012) as well as integrative conflict resolution (Rahim and Magner, 1995).

A fourth limitation of our study refers to the role of emotion regulation and how it is thought to translate into leaders’ communication behaviors. Specifically, we exclusively referred to the regulation of unpleasant emotions, while ignoring positive emotions. However, even though research on mindfulness and emotion regulation (for an overview, see Ostafin et al., 2015) has a strong focus on unpleasant emotions, such as anger, fear, or avoidance, from the perspective of Buddhism, also pleasant emotions, such as pride or desire, can be disturbing (Chambers et al., 2009). That said, mindful leaders should not only stay calm when unpleasant emotions arise but also in the presence of pleasant emotions. Thus, it will be interesting in future research on mindfulness in communication to give a stronger focus on the interplay and regulation of both pleasant and unpleasant emotional states. Such studies could include direct measures of specific emotion regulation strategies, most notably expressive suppression and cognitive reappraisal (Gross and John, 2003), and test whether and to what extent they may exert differential effects on mindfulness in communication.

In terms of more general directions for future research, it will be useful to replicate our results in different settings, such as mentoring or coaching relationships. In such studies it would be interesting to include alternative, more differentiated mindfulness scales which assess different facets of mindfulness (for a review see Sauer et al., 2013b). Although the Freiburg Mindfulness Inventory is a well-established instrument which is currently available in various languages (Sauer et al., 2013a), the use of other instruments such as the Five Factors Mindfulness Questionnaire (FFMQ) (Bohlmeijer et al., 2011; de Bruin et al., 2012) or the Comprehensive Inventory of Mindfulness Experiences (CHIME) (Bergomi et al., 2014), which measure different sub-facets of mindfulness, may help to further clarify the effects of mindfulness on communication behaviors. Notably, by further investigating the utility of the newly developed mindfulness in communication measure across different samples and contexts, and by comparing it to more nuanced measures of dispositional mindfulness, future research may address the call for alternative, “indirect” measures of a person’s level of mindfulness, which is grounded in the ongoing criticism of self-assessment questionnaires (e.g., Grossman, 2008). Although the validation of our newly developed instrument is still at an early stage, our study offers a promising basis for such indirect measures of dispositional mindfulness. In other words, measuring mindfulness in communication may aid future research in addressing the question of whether there are “objective and observable criteria of mindfulness” (Grossman, 2008, p. 407).

Another interesting avenue for future research could be to examine the cognitive processes behind mindfulness in communication in more detail. This is particularly true for the role of the capacity to disengage the self from the event, as reflected in the notion of *reperceiving* (Shapiro et al., 2006) or *decentering* (Hayes et al., 2004). Future studies could include a separate decentering measure (Fresco et al., 2007) and explore whether there are distinct relationships with the features of mindfulness in communication. Such studies may also benefit from more thoroughly disentangling the process of decentering. Recent research suggests that this kind of perspective shifting may be better understood as a process, including meta-awareness, disidentification from inner experience, and reduced reactivity to thought content (see Bernstein et al., 2015).

Finally, mindfulness research in general could benefit from taking up the reflections and criticisms of several scholars (Bodhi, 2011; Dreyfus, 2011; Purser and Milillo, 2015) who advocate a notion of mindfulness that goes beyond its current conceptualization in Western psychology.

### Practical Implications

Our focus on interpersonal benefits of mindfulness points to several practical implications, especially with regard to leadership development. While a large body of research on mindfulness-based interventions provides evidence that mindfulness can be trained (for meta-analyses see Grossman et al., 2004; Chiesa and Serretti, 2009; Cavanagh et al., 2014), research on mindfulness interventions in the workplace is still in its infancy. However, in practice, there is already a growing interest in mindfulness-based training programs, and many organizations presently use mindfulness-based trainings in personnel and leadership development (for examples see Marturano, 2010; Tan, 2012). This interest of practitioners is accompanied, and partly caused, by a growing body of non-scientific, popular literature, and a number of articles in newspapers and magazines, praising the benefits of a “mindful leadership style” (e.g., Caroll, 2008; Boyatzis and McKee, 2014). However, such reports are often grounded in anecdotal evidence and more rigorous research is needed to explore the role of mindfulness in the leadership context and to provide evidence-based approaches for practitioners in organizations. The findings of our study provide preliminary empirical support for the potential value of fostering mindfulness in organizations and suggest that mindfulness may not just promote personal wellbeing and resilience, as it has been shown by other scholars before, but also may have positive effects on interpersonal skills and communication behavior. Thus, since communication competencies are key to effective leadership, mindfulness-based interventions and training may represent a promising tool for effective leadership development. Despite the promising value of such leadership trainings, it is, however, important to consider potential pitfalls of mindfulness too. For instance, it is conceivable that a leader may use mindful communication for the mere purpose of impression management with selfish or unhealthy goals in mind (Reb et al., 2015b). An ethically informed view on corporate mindfulness, as advocated by several scholars in the last years (Purser and Milillo, 2015), may help to prevent potential dark side-effects of mindfulness.

As a general note of caution, it should be noted that mindfulness interventions in the workplace are not without risks. Several studies have shown that some participants may experience mindfulness interventions and related outcomes as challenging and distressing (Cebolla et al., 2017; Lindahl et al., 2017). One should generally not see mindfulness as a panacea for all sorts of challenges and problems leaders (and followers) are facing in their organizational practice. Mindfulness interventions can be useful if they are conducted by experts and carefully tailored to the needs and individual requirements of the participants. Furthermore, as Purser (2018) pointed out, the trend of mindfulness interventions at work can also be problematic because it tends to focus exclusively on the individual when it comes to cope with stress, instead of changing tasks or thinking about job design.

## Conclusion

By identifying mindfulness in communication as a behavioral manifestation of leaders’ dispositional mindfulness which mediates the latter’s relationship with followers’ satisfaction, our study provides a valuable contribution to the increasing body of literature on mindfulness in the workplace. Hopefully, it will stimulate more research on the role of mindfulness in communication behavior and in organizational contexts in general.

## Ethics Statement

The authors certify that the research presented in this manuscript has been conducted within the DGPs (German Psychological Society) ethical standards regarding research with human participants and scientific integrity.

## Author Contributions

JA and APV contributed conception and design of the study. JA contributed the acquisition of participants and data collection, and wrote the first draft of the manuscript. JA, APV, and KK performed the statistical analysis, wrote sections of the manuscript, and contributed to manuscript revisions, read, and approved the submitted version.

## Conflict of Interest Statement

The authors declare that the research was conducted in the absence of any commercial or financial relationships that could be construed as a potential conflict of interest. The reviewer MV and handling Editor declared their shared affiliation at the time of review.

## Footnotes

1 For exploratory purposes, we examined the difference between leaders who indicated practicing some form of mindfulness meditation (*n* = 10) and leaders who reported not practicing any form of mindfulness meditation (*n* = 24) with regard to their average dispositional mindfulness scores. Results showed that there was a significant difference with higher scores in the meditation group (*M* = 4.56, *SD* = 0.73) compared to the non-meditation group (*M* = 3.87, *SD* = 0.81), *t*(32) = 2.34, *p* < 0.05, *d* = 0.88.
